# COVID-19, Social Isolation, and Mental Health Among Older Adults: A Digital Catch-22

**DOI:** 10.2196/21864

**Published:** 2021-05-06

**Authors:** Theodore D Cosco, Karen Fortuna, Andrew Wister, Indira Riadi, Kevin Wagner, Andrew Sixsmith

**Affiliations:** 1 Gerontology Research Center Simon Fraser University Vancouver, BC Canada; 2 Oxford Institute of Population Ageing University of Oxford Oxford United Kingdom; 3 Dartmouth College Hanover, NH United States; 4 STAR Institute Simon Fraser University Vancouver, BC Canada

**Keywords:** social isolation, mental health, COVID-19, technology, older adult, psychology, digital health

## Abstract

One of the most at-risk groups during the COVID-19 crisis is older adults, especially those who live in congregate living settings and seniors’ care facilities, are immune-compromised, and/or have other underlying illnesses. Measures undertaken to contain the spread of the virus are far-reaching, and older adults were among the first groups to experience restrictions on face-to-face contact. Although reducing viral transmission is critical, physical distancing is associated with negative psychosocial implications, such as increased rates of depression and anxiety. Promising evidence suggests that participatory digital co-design, defined as the combination of user-centered design and community engagement models, is associated with increased levels of engagement with mobile technologies among individuals with mental health conditions. The COVID-19 pandemic has highlighted shortcomings of existing technologies and challenges in their uptake and usage; however, strategies such as co-design may be leveraged to address these challenges both in the adaptation of existing technologies and the development of new technologies. By incorporating these strategies, it is hoped that we can offset some of the negative mental health implications for older adults in the context of physical distancing both during and beyond the current pandemic.

Older adults constitute one of the most at-risk groups during the ongoing COVID-19 crisis, especially those who live in congregate living settings and seniors’ care facilities, are immune-compromised, and/or have other underlying illnesses [1]. Measures undertaken to contain the spread of the virus are far-reaching, and older adults were among the first groups to have experienced restrictions with regard to face-to-face contact. Although reducing viral transmission is critical, physical distancing is known to have significant negative psychosocial implications, such as increased rates of depression and anxiety, particularly for older adults [2].

Apart from the COVID-19 outbreak, older adults, in general, experience the highest rates of social isolation and loneliness, which are associated with a range of negative mental health outcomes [3]. Physical distancing measures may have negative impacts on older adults living in the community, especially for those who rely on formal or informal support and care from family and/or friends, those who utilize religious or community centers as social hubs, as well as those who live in supportive or assisted living centers (where gathering in common spaces used for meals and other activities must cease as a result of physical distancing measures). This double burden of age-related diminishing social spheres and the implementation of measures that require physical distancing and enforced isolation presents significant mental health risks for older people.

In response to “stay at home” orders, sweeping transitions to web-based communication platforms have been adopted as a means of fostering and maintaining social connectedness; however, older adults use these technologies at disproportionately lower rates than younger individuals, both during and beyond the COVID-19 pandemic. This digital divide, which involves barriers to digital technologies [4], is a major inhibitive force in facilitating digital social connectedness among older adults while following the stay-at-home orders. The first level of the digital divide includes barriers to uptake and access to technology (eg, financial limitations), whereas the second level concerns barriers to the usage of these technologies (eg, functional impairments in manual dexterity) [5]. Most videoconferencing or communication platforms have not been developed with the older adult in mind, thereby leading to low rates of uptake before the COVID-19 pandemic and difficulty in accessing and using these platforms during the pandemic.

Consequently, we find ourselves in a catch-22 situation where negative mental health implications of social isolation and loneliness among older adults could potentially be mitigated by the use of digital solutions, but only if the person already has the technological knowledge, desire, and access to use these technologies. Furthermore, without the capacity to interact face-to-face with older adults during the COVID-19 outbreak, it can be challenging, albeit possible, to provide guidance on how to use technologies, an issue further compounded by physiological challenges (eg, reduced visual acuity, manual dexterity, and cognitive impairment). Despite its huge potential, the impact of implementing technologies to reduce social isolation and loneliness has been limited in terms of real-world products and services [6]. Unfortunately, it is often the individuals who would benefit most from digital solutions who are the most vulnerable and least likely to access these technologies. Moving forward, there is a need for a major shift in research culture away from a researcher- and technology-driven agenda to one that focuses on real-world problems and solutions [6,7]. The digital divide spans a considerable breadth of issues within the context of the pandemic; therefore, we will focus on the challenge of technology development that is not inclusive of older adults’ perspectives.

In the short term, we must aim to foster the uptake of existing technologies through trainings designed for older adults and address normal age-related changes that impede technology use (ie, changes to memory, eyesight, or mobility), with longer-term objectives to incorporate older adults throughout the software development lifecycle of new technologies. Within the context of the current challenges presented by physical distancing, repurposing or reframing existing technologies may be effective in mitigating social isolation and feelings of loneliness among the older population. For example, the telephone may not be the leading edge of innovation, but a landline phone call is a demonstrated and effective means of communication and human connection [8]. With respect to new technologies—that may be critical in addressing future pandemics—incorporating digital peer support is one of the best ways to foster technology engagement [9]. Through these live or automated peer support services that can be delivered through various digital platforms, such as peer-to-peer networks on social media or peer-delivered interventions supported with smartphone apps, older adults can develop the tools needed to actively engage with technology. The similarity, bond, and trust within this relationship supports reciprocal accountability and engagement, which may promote engagement as older adults model and teach others how to use technology. For example, older adult peer support specialists with lived experience of aging with a mental health condition (and commonly a physical health condition), who are trained and accredited by their respective state to provide Medicaid and/or Medicare reimbursable telehealth services, can use their personal experiences to help address challenges of normal aging and technology use (eg, changes to eyesight, fine motor skills, and hearing). Through such support from individuals with lived experiences of aging, it is hoped that greater technology use and uptake can be fostered.

Promising evidence suggests that participatory digital co-design, defined as the combination of user-centered design and community engagement models, shows high levels of engagement with mobile technologies among individuals with mental health conditions [9]. The COVID-19 pandemic has certainly highlighted the shortcomings of existing technologies and challenges in their uptake and usage [10]; however, strategies such as co-design may be leveraged to address these challenges both in the adaptation of existing technologies and the development of new technologies.

In populations where individuals are not living with mental illness, participatory digital co-design has led to promising developments with the potential for more relevant research with wider impact, better internal validity, and rapid translation of research into practice—and greater engagement [9]; therefore, it has the potential to reduce the digital divide [11]. By incorporating the aforementioned strategies, it is hoped that we can offset some of the negative mental health implications for older adults in the context of physical distancing, both during and beyond the COVID-19 pandemic.

## References

[1] Zhou F, Yu T, Du R, Fan G, Liu Y, Liu Z, Xiang J, Wang Y, Song B, Gu X, Guan L, Wei Y, Li H, Wu X, Xu J, Tu S, Zhang Y, Chen H, Cao B (2020). Clinical course and risk factors for mortality of adult inpatients with COVID-19 in Wuhan, China: a retrospective cohort study. Lancet.

[2] Robb CE, de Jager CA, Ahmadi-Abhari S, Giannakopoulou P, Udeh-Momoh C, McKeand J, Price G, Car J, Majeed A, Ward H, Middleton L (2020). Associations of social isolation with anxiety and depression during the early COVID-19 pandemic: A survey of older adults in London, UK. Front Psychiatry.

[3] Coyle CE, Dugan E (2012). Social isolation, loneliness and health among older adults. J Aging Health.

[4] Levy H, Janke AT, Langa KM (2015). Health literacy and the digital divide among older Americans. J Gen Intern Med.

[5] Gell NM, Rosenberg DE, Demiris G, LaCroix AZ, Patel KV (2015). Patterns of technology use among older adults with and without disabilities. Gerontologist.

[6] Sixsmith A, Mihailidis A, Simeonov D (2017). Aging and technology: Taking the research into the real world. Public Policy & Aging Report.

[7] Cosco TD, Firth J, Vahia I, Sixsmith A, Torous J (2019). Mobilizing mHealth data collection in older adults: Challenges and opportunities. JMIR Aging.

[8] Van Orden KA, Bower E, Lutz J, Silva C, Gallegos AM, Podgorski CA, Santos EJ, Conwell Y (2020). Strategies to promote social connections among older adults during 'social distancing' restrictions. Am J Geriatr Psychiatry. Epub ahead of print posted online on May 18, 2020.

[9] Fortuna KL, Naslund JA, LaCroix JM, Bianco CL, Brooks JM, Zisman-Ilani Y, Muralidharan A, Deegan P (2020). Digital peer support mental health interventions for people with a lived experience of a serious mental illness: Systematic review. JMIR Ment Health.

[10] Moore RC, Hancock JT (2020). Older Adults, Social Technologies, and the Coronavirus Pandemic: Challenges, Strengths, and Strategies for Support. Social Media + Society.

[11] Unertl K, Schaefbauer C, Campbell T, Senteio C, Siek K, Bakken S, Veinot TC (2016). Integrating community-based participatory research and informatics approaches to improve the engagement and health of underserved populations. J Am Med Inform Assoc.

